# The Impact of Preclinical High Potent P2Y_12_ Inhibitors on Decision Making at Discharge and Clinical Outcomes in Patients with Acute Coronary Syndrome

**DOI:** 10.3390/jcm12124094

**Published:** 2023-06-16

**Authors:** Andreas Hammer, Mario Krammel, Patrick Aigner, Georg Pfenneberger, Sebastian Schnaubelt, Felix Hofer, Niema Kazem, Lorenz Koller, Eva Steinacher, Ulrike Baumer, Christian Hengstenberg, Alexander Niessner, Patrick Sulzgruber

**Affiliations:** 1 Division of Cardiology, Department of Internal Medicine II, Medical University of Vienna, 1090 Vienna, Austria; andreas.hammer@muv.ac.at (A.H.); christian.hengstenberg@muv.ac.at (C.H.);; 2 Emergency Medical Service of Vienna, 1030 Vienna, Austria; mario.krammel@wien.gv.at (M.K.); patrik.aigner@wien.gv.at (P.A.); georg.pfenneberger@wien.gv.at (G.P.); 3 Department of Emergency Medicine, Medical University of Vienna, 1090 Vienna, Austria

**Keywords:** P2Y12 inhibitor loading, acute coronary syndrome, ticagrelor, prasugrel, STEMI, cardiovascular mortality, re-PCI

## Abstract

Background: Purinergic signaling receptor Y_12_ (P2Y_12_) inhibitors are a fundamental part of pharmacological therapy in acute coronary syndrome (ACS) for preventing recurrent ischemic events. Current guidelines support the use of prasugrel over ticagrelor—however, ticagrelor is widely used for preclinical loading during ACS due to its ease of administration. In this regard, it remains unknown whether the preclinical loading with P2Y_12_ inhibitors impacts decision-making for the long-term dual antiplatelet strategy, as well as cardiovascular outcomes, including re-percutaneous coronary intervention in real-world settings. Methods: Within this population-based prospective observational study, all patients with ACS who received medical care via the Emergency Medical Service (EMS) in the city of Vienna between January 2018 and October 2020 were enrolled. Patients were stratified according to their P2Y_12_ inhibitor loading regimen. Subsequently, the association of P2Y_12_ inhibitor loading on long-term prescription at discharge and outcome was assessed. Results: The entire study cohort consisted of 1176 individuals with ST-elevation myocardial infarction (STEMI), of whom 47.5% received prasugrel and 52.5% ticagrelor. The likelihood of adhering to the initial P2Y_12_ inhibitor strategy during the clinical stay was high for both ticagrelor (84%; OR: 10.00; *p* < 0.001) and prasugrel (77%; OR: 21.26; *p* < 0.001). During patient follow-up (median follow-up time three years), 84 (7.1%) patients died due to cardiovascular causes, and 82 (7.0%) patients required re-PCI. Notably, there was no difference in cardiovascular mortality (6.6% ticagrelor vs. 7.7% prasugrel) or re-PCI rates (6.6% ticagrelor vs. 7.3% prasugrel) addressing the P2Y_12_ inhibition strategy. Conclusion: We observed that, regardless of the initial antiplatelet inhibitor strategy, the in-hospital P2Y_12_ adherence was exceedingly high, and there was a minimal occurrence of switching to another P2Y_12_ inhibitor. Most importantly, no significant difference in cardiovascular death/re-PCI between ticagrelor and prasugrel-based preclinical loading has been observed. Consequently, the choice of high potent P2Y_12_ did not influence the cardiac outcome from a long-term perspective.

## 1. Introduction

Within recent years, the management of ST-elevation myocardial infarction (STEMI) has substantially evolved through the emergence of percutaneous coronary intervention (PCI) together with concomitant antithrombotic therapy [1]. The latter is a crucial cardioprotective factor in various stages of acute coronary syndrome (ACS), which ranges from preclinical loading (pre-hospitalization) to long-term post-interventional therapy. It is well known that patients affected by ACS possess a greater risk of subsequent ischemic events, which increases mortality with every additional incident [2]. In this regard, platelet aggregation inhibitors via fast-acting purinergic signaling receptor Y_12_ (P2Y_12_) inhibitors (i.e., clopidogrel, ticagrelor, prasugrel) are a fundamental part of the pharmacological therapy of ACS [3]. Ticagrelor and prasugrel provided a more potent antiplatelet inhibition compared to clopidogrel and were found to be superior in preventing ischemic events [4,5,6,7].

Usually, STEMI patients will receive pre-interventional loading with aspirin (150–300 mg p.o. or 75–250 mg i.v. if no aspirin is already prescribed) in combination with a P2Y_12_ inhibitor, preferred 60 mg prasugrel or 180 mg ticagrelor or if contraindications are present 600 mg clopidogrel [8]. So far, prasugrel and ticagrelor were considered equal in efficacy and thus interchangeable in ACS patients; however, the ISAR-REACT 5 trial indicated that a prasugrel-based DAPT showed superior outcome compared to a ticagrelor-based approach [9]. Precisely, a lesser extent of the composite endpoint (myocardial infarction, ischemic stork, and death) was present in the prasugrel-treated DAPT arm, which was predominantly caused by decreased events of reoccurring myocardial ischemic events (prasugrel-DAPT: 6.9% vs. ticagrelor-DAPT: 9.3%; *p* = 0.006) [9]. Despite that, a non-inferiority in major bleeding events compared to ticagrelor was observed [9]. Notably, the outcome of ISAR-REACT 5 encompassed all patients with ACS rather than exclusively focusing on STEMI cases. According to this data, a prasugrel-based DAPT approach in ACS patients appears to be beneficial compared to the combination of ticagrelor and aspirin, which was subsequently adopted by current guidelines of the ESC. Nonetheless, ISAR-REACT 5 had its limitations, primarily due to its open label design. Additionally, notable disparities were observed in the percentage of excluded patients between the treatment arms, with 11.6% in the prasugrel group compared to 1.14% in the ticagrelor group, particularly concerning the safety analysis. Recently, there have been certain differences in the galenic formulation of the two antiplatelet agents available, which becomes an important factor during preclinical P2Y_12_ inhibitor loading. Ticagrelor offers an orodispersible tablet that favors simple administration compared to standard prasugrel tablets [10]. It is noteworthy that in critically ill patients, including a significant proportion of those with ACS, the use of oral pharmacological agents requiring water for pill swallowing may not be an ideal route of drug administration. This is particularly relevant in time-sensitive settings such as ambulances, emergency departments, or catheterization laboratories, where rapid action is essential for effective patient management. Prompt access to water for swallowing pills may be limited or technically challenging in these circumstances. Therefore, the utilization of orally disintegrating tablets offers a safe, effective, and convenient alternative method for administering ticagrelor to ACS patients [10]. Nevertheless, according to current guidelines, both prasugrel and ticagrelor receive equal recommendations for treatment in STEMI patients [8]. In contrast, in individuals presenting with non-ST-segment elevation myocardial infarction (NSTEMI), a prasugrel-based approach is favored [3]. However, data on whether the choice of P2Y_12_ inhibition during preclinical loading for ACS impacts patient outcome from a long-term perspective remain scarce and inconclusive.

Therefore, this study aimed to examine real-world differences in preclinical P2Y_12_ inhibitor loading prescription in STEMI patients and whether long-term outcome and re-PCI rates are affected by the individual approach within this unselected patient population.

## 2. Methods

### 2.1. Study Design and Setting 

Within the present population-based prospective observational study, all patients presenting with STEMI and receiving medical care via the Emergency Medical Service (EMS) of the city of Vienna between January 2018 and October 2020 were investigated. STEMI was defined in accordance with current guidelines of the European Society of Cardiology (ESC) [3].

**Figure 1:**
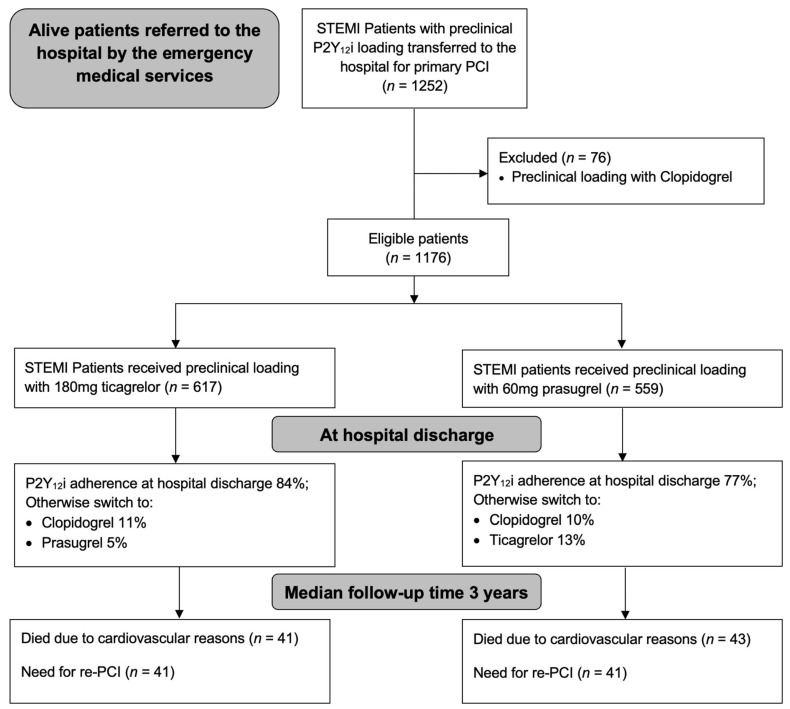
Study design and outcome illustrated as a flow chart. Purinergic signaling receptor Y12 inhibitors (P2Y_12_i); ST-elevation myocardial infarction (STEMI); Re-percutaneous coronary intervention (re-PCI).

As part of a population-based analysis, there were no specific exclusion criteria for patient enrollment, except preclinical loading with clopidogrel. The study protocol complied with the declaration of Helsinki and was approved by the local ethics committee of the Medical University of Vienna (EK 159/2011). Figure 1 presents the study design in the form of a flowchart.

### 2.2. System Description of Preclinical Loading with P2Y_12_ Inhibitors

In the case of STEMI, a preclinical loading of Aspirin and a P2Y_12_ inhibitor is conducted via emergency physicians. Between 1/2018 and 9/2019, 250–500 mg aspirin (intravenous) and 60 mg prasugrel (oral) were used as standardized loading regimens. Since 9/2019, the therapeutic approach was adapted to 250–500 mg aspirin (intravenous) and 180 mg ticagrelor (as an orodispersible tablet). This adjustment was implemented in response to the availability of an orodispersible galenic formulation, which facilitated easier preclinical administration of the medication.

### 2.3. Data Acquisition and Definition

Patient-relevant characteristics were assessed at the time of clinical presentation during the first contact with paramedics or an emergency physician of the emergency medical service (EMS) of the city of Vienna. Data were obtained via a standardized electronic protocol for event documentation as part of the quality management and inserted into a predefined electronic record abstraction form. The presence of STEMI was diagnosed by trained paramedics and validated by emergency physicians. Data for consecutive patients with STEMI receiving medical care via EMS were analyzed and validated in an anonymized fashion by specially trained chart reviewers. Data were double-checked for consistency by study investigators. Data on mortality were assessed via screening the national registry of death (Austrian Registry of Death, Statistics Austria, Vienna, Austria) until October 2022. Subsequently, re-PCI rates were evaluated through a search of the Vienna Healthcare Group hospitalizations database (“Wiener Gesundheitsverbund”). All outcomes were defined according to the International Classification of Diseases, Tenth Revision (ICD-10). A composite of cardiovascular mortality and re-PCI was chosen as the endpoint for the long-term follow-up.

### 2.4. Statistical Analysis

Continuous data are shown as median and interquartile range (IQR) and compared using the Kruskal-Wallis test. Categorical parameters are presented as counts and percentages and analyzed using the Chi-square test. Univariate regression analyses determined the probability of P2Y_12_ inhibitor regimen adherence at discharge in each subgroup. 3-year Kaplan-Meier event rates were compared using the log-rank test. Cox regression analysis was used to assess the impact of preclinical loading on the combined endpoint of cardiovascular death/re-PCI. The results are presented as hazard ratio (HR), including their respective 95% confidence interval (CI). Statistical significance was defined by two-sided *p*-values < 0.05. Statistical analyses were performed using SPSS 26.0 (IBM SPSS, Armonk, NY, USA).

## 3. Results

A detailed summary of the baseline characteristics can be found in Table 1. In brief, the entire study cohort consisted of 1176 individuals with STEMI. The participants were predominantly male (75.1%; *n* = 883), and the median age was 60 (IQR 52.6–69.0) years. Patients were stratified into two separate cohorts, according to their date of study inclusion (either between A: 1 January 2018–31 August 2019 or B: 1 September 2019–1 October 2020).

The latter cohort included patients with worse eGFR levels (44.8 mg/dL; IQR 37.8–55.1; *p* = 0.007), whereas the former contained more male (*n* = 485; 78.5%; *p* = 0.005) patients. Furthermore, preclinical P2Y_12_ inhibitor loading differed between the groups due to protocol alteration over the observed period. Between the period from 1 January 2018–31 August 2019, significantly more preclinical loading with prasugrel (*n* = 556; 90.0%; *p* < 0.001) was observed. While from 1 September 2019–1 October 2020, ticagrelor (*n* = 555; 99.5%; *p* < 0.001) was the predominant preclinically administered P2Y_12_ inhibitor.

### 3.1. Adherence to Initial P2Y_12_ Inhibitor during Hospital Stay 

**Figure 2:**
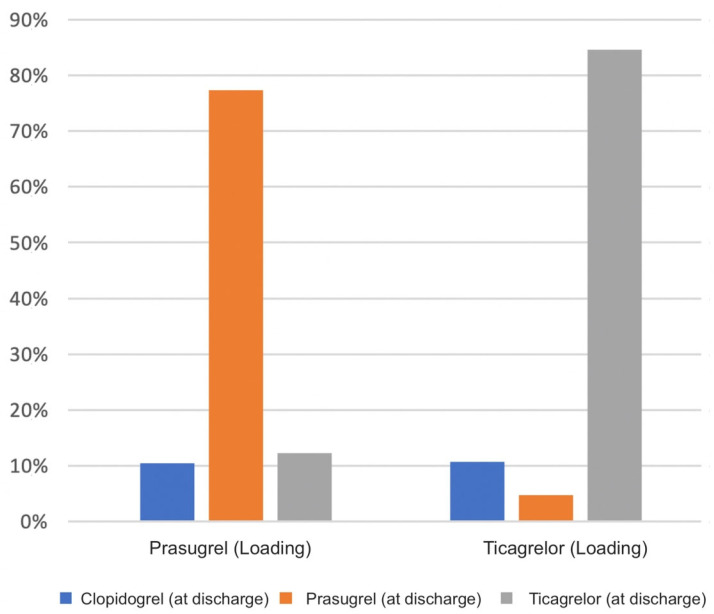
Patient proportion that adhered or moved to another P2Y_12_ inhibitor throughout the hospital stay in relation to initial P2Y_12_ inhibitor therapy. Prasugrel: 77% stayed on prasugrel, 13% were switched to ticagrelor and 10% to clopidogrel. Ticagrelor: 84% stayed on this treatment approach, 11% were switched to clopidogrel, and 5% to prasugrel.

Figure 2 visualizes the patient proportion who adhered or moved to another P2Y_12_ inhibitor throughout the hospital stay in relation to initial P2Y_12_ inhibitor therapy. We observed that 84% of the individuals who initially received ticagrelor remained on this treatment approach, 11% were switched to clopidogrel, and 5% to prasugrel. A similar trend was observed with prasugrel-loaded patients. 77% stayed on prasugrel, whereas 13% were switched to ticagrelor and 10% to clopidogrel. The likelihood (represented as OR) of keeping the same loading P2Y_12_ inhibitor was assessed by comparing loading strategy and medication at discharge within a regression analysis. For patients who received prasugrel, a statistically significant crude OR of 21.26 (CI95% 10.25–44.08; *p* < 0.001) was found (Table 2). Subsequently, the same analysis was performed for ticagrelor, revealing similar significant results OR 10.00 (CI95% 6.08–16.45; *p* < 0.001).

### 3.2. The Impact of P2y_12_ Inhibitor Loading on Patient Outcome

Table 3 illustrates the entire cohort stratified by the different preclinical highly potent P2Y_12_ inhibitor administration. Overall, ticagrelor (*n* = 617; 52.5%) was the most frequently used P2Y_12_ inhibitor, followed by prasugrel (*n* = 559; 47.5%) within the total observation period. In addition, the ticagrelor subgroup showed significantly worse eGFR levels with 45.5 mg/min/1.73m^2^ (IQR: 37.8–55.1) as compared to the fraction receiving prasugrel 56.1 mg/dL (IQR: 41.9–59.0); *p* = 0.004). No statistical differences in cardiovascular mortality (6.6% ticagrelor vs. 7.7% prasugrel) or re-PCI rates (6.6% ticagrelor vs. 7.3% prasugrel) have been observed between the groups.

**Figure 3:**
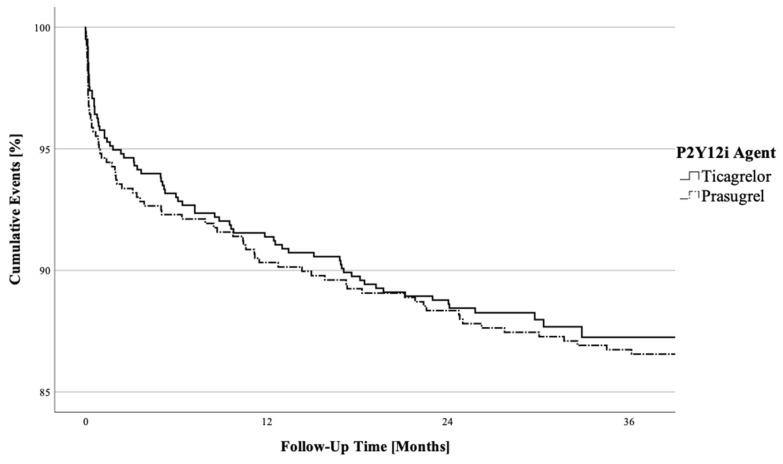
Kaplan-Meier curves for cardiovascular mortality/re-PCI stratified by initial P2Y_12_ inhibitor loading approach. Observed event rates were compared using the log-rank test, *p* = 0.746. The solid line indicates ticagrelor, and the dotted line is the prasugrel subgroup.

Among the patients who underwent follow-up for a median duration of 1076 days (with an interquartile range of 838–1424 days), 84 individuals (7.1%) died due to cardiovascular reasons. Additionally, 82 (7.0%) patients had a subsequent coronary event that required re-PCI. As illustrated in Figure 3, Kaplan—Meier survival curves were plotted for the combined endpoint cardiovascular mortality/re-PCI. The curves for both loading approaches appeared comparable, showing at the end of the observation period an overall survival of 87.1% in initial ticagrelor and 86.5% in prasugrel receivers. Comparing the two outcome rates via a log-rank test, no statistical significance (*p* = 0.746) was found. Moreover, within a Cox regression model, no association between initial P2Y_12_ inhibitor therapy and outcome was observed (HR 1.01 (CI95% 0.86–1.19) *p* = 0.869) (Table 4).

## 4. Discussion

Within this unselected real-world analysis, it was observed that there is a high probability that the preclinical P2Y_12_ inhibitor loading approach will determine the subsequent choice of P2Y_12_ inhibition during long-term DAPT. Most importantly, there was no difference in clinical outcomes when comparing STEMI patients who received ticagrelor to prasugrel recipients.

The role of optimal antiplatelet therapy in STEMI patients undergoing primary PCI is crucial to prevent further myocardial ischemic events and thus substantially influence mortality. Within this regard, several studies have compared different dosages regimen and efficacy levels of P2Y_12_ inhibitors. Early oral administration of 150 to 300 mg (or i.v. if not able to swallow) acetylsalicylic acid serves as a foundation through the inhibition of prostacyclin synthesis in thrombocytes [3]. Additionally, it is recommended to induce anticoagulation with heparin and establish DAPT by administration of a high-dose P2Y_12_ inhibitor before primary PCI [3,8]. Concerning the latter, there is clear evidence that highly potent P2Y_12_ inhibitors (i.e., ticagrelor and prasugrel) outperform clopidogrel in efficacy by reducing ischemic, thromboembolic events, and cardiovascular death [4,5,6]. However, some studies reported an elevated risk of bleeding, and thus clopidogrel is still utilized among high-bleeding-risk patients [3,8]. The randomized Study of Platelet Inhibition and Patient Outcomes (PLATO) has investigated the efficacy of ticagrelor (180 mg loading, 90 mg twice daily maintenance dose) or clopidogrel (300–600 mg loading, followed by 75 mg daily) combined with aspirin (325 mg loading, 75–100 mg daily; 325 mg daily after stent placement for 6 months allowed, 54% of US patients) in 18,624 ACS patients [7]. Both study cohorts consisted of about 40% NSTEMI and over one-third of STEMI patients who received primary PCI (~60%) or coronary-artery bypass graft (CABG) (~10%). The authors concluded that ticagrelor was superior in the primary endpoints (stroke, myocardial infarction, cardiovascular death) than clopidogrel while being non-inferior in observed major bleedings. Nevertheless, higher events of non-procedure-related bleedings were observed within the ticagrelor group [5]. Furthermore, more participants who received ticagrelor developed short-lasting dyspnea episodes, which resulted in 0.9% of drug discontinuation in this study arm. In a subgroup analysis of the PLATO trial, which involved 7544 patients diagnosed with STEMI and scheduled for primary PCI, consistent findings aligned with the main trial results [11]. The authors reported a reduction in the composite endpoint of stroke, MI, and cardiovascular death in patients treated with ticagrelor compared to clopidogrel (9.4% vs. 10.8%; *p* = 0.07) [11]. Importantly, this benefit was observed without an increased risk of major bleeding events (9.0% vs. 9.2%; *p* = 0.76) [11].

The randomized Trial to Assess Improvement in Therapeutic Outcomes by Optimizing Platelet Inhibition with Prasugrel-Thrombolysis in Myocardial Infarction (TRITON-TIMI 38) compared the two different DAPT regimens of prasugrel (loading dose 60 mg, maintenance dose 10 mg) or clopidogrel (loading dose 300 mg, maintenance 75 mg) with concomitant aspirin in 13,608 ACS patients with scheduled PCI [6]. The prasugrel arm showed superior efficacy over clopidogrel in primary endpoints (cardiovascular death, stroke, MI). Of specific note, incidence rates of stent thrombosis were clearly lower with prasugrel compared to clopidogrel (1.1% vs. 2.4% *p* = < 0.001). This effect was observed as early as day 3 and persisted through the entire observation period of 15 months. However, this superior efficacy came at the price of an excess of TIMI major and minor bleedings (TIMI major bleeding: 2.4% prasugrel vs. 1.8% clopidogrel, *p* = 0.03; TIMI minor bleeding: 5.0% prasugrel vs. 3.8% clopidogrel *p* = 0.002) [6]. A subgroup analysis conducted within the TRITON trial, focusing on 3514 patients diagnosed with STEMI, also reported a risk reduction in the prasugrel arm compared to clopidogrel for the primary outcome (HR 0.79; *p* = 0.022) [12]. This risk reduction was primarily driven by lower rates of MI (6.8% prasugrel vs. 9.0% clopidogrel). Additionally, the incidence of stent thrombosis was again significantly lower in the prasugrel group compared to clopidogrel (1.6% vs. 2.8%). In contrast to the main trial findings, there were no significant differences between the two treatment arms regarding TIMI major bleeding (2.4% prasugrel vs. 2.1% clopidogrel, *p* = 0.645) or TIMI major or minor bleeding (5.1% prasugrel vs. 4.7% clopidogrel, *p* = 0.649) [12].

Since there was now sufficient evidence that prasugrel and ticagrelor showed greater efficacy than clopidogrel, The Intracoronary Stenting and Antithrombotic Regimen: Rapid Early Action for Coronary Treatment (ISAR-REACT 5) trial recently investigated the efficacy levels among these two high potent P2Y_12_ inhibitors in 4018 ACS individuals scheduled for coronary angiography [9]. The cohorts were stratified into a prasugrel (60 mg loading dose, 10 mg maintenance dose; if >70 years or <60 kg, reduced maintenance dose of 5 mg) and ticagrelor (180 mg loading dose, 90 mg maintenance dose) treatment arm with consecutive aspirin. Overall, 83% of the participants received PCI, and about 40% were diagnosed with STEMI. The authors concluded that the DAPT regimen based on prasugrel was superior to a ticagrelor-based DAPT, showing a reduced incidence of the primary endpoint that is composed of death, stroke, MI (6.9% prasugrel-DAPT vs. 9.3% ticagrelor-DAPT; *p* = 0.006, NNT = 42), which was predominantly favored by a substantial reduction of MI [9]. Moreover, the safety endpoint (BARC type 3 to 5 bleeding) showed no statistical differences among the study arms (4.8% prasugrel-DAPT vs. 5.4% ticagrelor-DAPT; *p* = 0.46). Of note, a ticagrelor-based approach led to less therapy adherence (12.5% prasugrel vs. 15.2% ticagrelor, *p* = 0.03), presumably due to ticagrelor specific side effects of dyspnea and the more intricately regimen (twice daily) [9]. In conclusion, the presented data of ISAR-REACT 5 indicate benefits in almost every relevant patient subgroup and on-treatment analysis. However, some concerns about study design and results have been raised by the scientific community. Within this regard, most study endpoints (83%) were only assessed via follow-up telephone visits which fosters detection bias. Furthermore, it appears that patients with a history of stroke have been excluded from ISAR-REACT 5, which could promote selection bias. Taking into consideration the outcomes of the TRITON-TIMI 38 and PLATO trials, it becomes apparent that prasugrel showed an increased incidence of bleeding events, while ticagrelor exhibited no adverse effects in the latter study involving the same group of patients. These observations raise the possibility that the exclusion of such individuals in the ISAR-REACT 5 trial may have influenced the safety and efficacy outcomes of prasugrel, suggesting a potentially lower overall performance.

An additional noteworthy concern in ISAR-REACT 5 pertains to the timing of antiplatelet loading. Patients without ST elevation assigned to ticagrelor received the loading dose immediately after randomization, whereas those assigned to prasugrel had their loading dose postponed until the coronary anatomy was determined. Therefore, this study compared the two drugs in the context of a loading strategy, despite this difference in timing.

The present analysis revealed a high probability that clinicians would adhere to the initial P2Y_12_ inhibitor agent, which was administered during a hospital transfer. Interestingly, we observed a greater therapy change from initial prasugrel to ticagrelor at discharge rather than vice versa (13% vs. 5%). The results of the ISAR-REACT 5 trial suggest that a prasugrel-based antithrombotic DAPT approach is superior to a ticagrelor-based approach in patients with ACS. However, within the present study, we found that patients receiving ticagrelor had comparable outcomes to those receiving prasugrel. In this regard, it is also important to consider that our study population included an unselected cohort of patients, which represents real-world clinical practice. In such a population, the efficacy of a treatment approach may not necessarily mirror the results of a randomized controlled trial or a highly selected population. Therefore, our findings suggest that the two treatment strategies may be equally effective. Another possibility is that our study was underpowered to detect a true outcome difference between the two treatment groups. The ISAR-REACT 5 trial included over 4000 patients—therefore, it is important to acknowledge that the present study may have been underpowered to detect a relevant difference in outcomes between treatment groups.

### Limitations

Several limitations to this study should be acknowledged. First, this study only included STEMI patients recognized via the Emergency Medical Service, which may limit the generalizability of the findings to other settings. Second, the sample size was relatively small, which may limit the statistical power to detect significant differences between the study groups in outcome. Third, because some participants may have received care outside of the study location, there is a possibility that relevant outcome variables may have been missed. Fourth, the follow-up data were obtained from electronic records rather than directly from the patients.

## 5. Conclusions

STEMI patients possess a greater risk of subsequent ischemic events, which increases mortality with every additional incident. Therefore, to prevent these adverse events, sufficient antithrombotic therapy is of utmost importance. According to current guidelines, both prasugrel and ticagrelor receive equal recommendations for treating STEMI patients. However, in the case of NSTEMI patients, a prasugrel-based approach is preferred based on evidence derived from the head-to-head comparison conducted in the ISAR-REACT 5 trial. Regarding practicality, ticagrelor holds an advantage in the preclinical setting due to the availability of an orodispersible galenic formulation. The present analysis observed that regardless of the initial antiplatelet inhibitor strategy, the in-hospital P2Y_12_ adherence was exceedingly high, and there were minimal instances of therapy switching. Interestingly, the choice of P2Y12 inhibitor did not demonstrate any significant influence on the incidence of cardiovascular death or re-PCI. Hence, the lack of observed differences in outcomes suggests that a ticagrelor-based approach may be as effective as prasugrel in an unselected population but warrant further investigation.

## Figures and Tables

**Table 1:** Baseline characteristics.

	Entire Cohort (*n* = 1176)	Period 1 (*n* = 618)	Period 2 (*n* = 558)	*p*-Value
Clinical Presentation				
Age, years (IQR)	60.0 (52.6–69.0)	60.0 (53.0–67.0)	60.0 (53.0–70.0)	0.263
Gender (male), *n* (%)	883 (75.1)	485 (78.5)	398 (71.3)	0.005
Comorbidities				
Diabetes, *n* (%)	181 (15.4)	88 (14.2)	93 (16.7)	0.142
CKD, *n* (%)	51 (4.3)	22 (3.5)	29 (5.2)	0.123
Prior MI, *n* (%)	461 (39.2)	250 (40.5)	211 (37.8)	0.721
Prior TIA/Stroke, *n* (%)	47 (4.0)	25 (4.0)	22 (3.9)	0.952
Loading Medication				
Prasugrel, *n* (%)	559 (47.5)	556 (90.0)	3 (0.5)	<0.001
Ticagrelor, *n* (%)	618 (52.6)	62 (10.0)	555 (99.5)	<0.001
Prior NOAC/VKA, *n* (%)	18 (1.5)	9 (1.5)	9 (1.6)	0.827
Laboratory variables				
NT–proBNP, pg/mL (IQR)	155.7 (52.6–561.7)	155.1 (44.5–556.1)	165.3 (56.3–606.7)	0.554
eGFR, mg/min/1.73 m^2^ (IQR)	52.6 (39.7–57.9)	55.8 (41.9–58.6)	44.8 (37.8–55.1)	0.007
Outcome variables				
Death, *n* (%)	84 (7.1)	45 (7.3)	39 (7.0)	0.976
Re–PCI, *n* (%)	82 (6.9)	43 (7.0)	39 (7.0)	0.507

Categorical data are presented as counts and percentages and analyzed using Chi-square-test. Continuous data are presented as median and the respective interquartile range and analyzed using the Mann-Whitney U test. Period 1 (1 January 2018–31 August 2019); Period 2 (1 September 2021–1 October 2020). ST-elevation myocardial infarction (STEMI); Chronic kidney disease (CKD); Myocardial infarction (MI); Transient ischemic attack (TIA); Non-vitamin K antagonist oral anticoagulant (NOAC); Vitamin-K antagonist (VKA); N-terminal pro b-type natriuretic peptide (NT-proBNP); Estimated glomerular filtration rate (eGFR); Re-percutaneous coronary intervention (re-PCI).

**Table 2:** Probability of the respective P2Y_12_ inhibitor at discharge after loading with the same antiplatelet agent.

	OR (95% CI)	*p*-Value
Prasugrel at discharge	21.26 (10.25–44.08)	<0.001
Ticagrelor at discharge	10.00 (6.08–16.45)	<0.001

The odds ratio of maintaining the initial P2Y12 inhibitor strategy at discharge within a regression analysis.

**Table 3:** Baseline characteristics stratified by loading medication.

	Ticagrelor (*n* = 617)	Prasugrel (*n* = 559)	*p*-Value
Clinical Presentation			
Age, years (IQR)	60.1 (52.9–70.1)	60.0 (53.0–67.0)	0.154
Gender (male), *n* (%)	442 (71.6)	441 (78.9)	0.004
Comorbidities			
Diabetes, *n* (%)	96 (15.6)	85 (15.2)	0.679
CKD, *n* (%)	31 (5.0)	20 (3.6)	0.179
Prior MI, *n* (%)	232 (37.6)	229 (40.9)	0.451
Prior TIA/Stroke, *n* (%)	24 (3.8)	23 (4.1)	0.939
Medication			
Prior NOAC/VKA, *n* (%)	9 (1.5)	9 (1.6)	0.833
Prior Clopidogrel, *n* (%)	2 (0.3)	4 (0.7)	0.357
Prior Ticagrelor, *n* (%)	1 (0.2)	0	0.341
Prior Prasugrel, *n* (%)	1 (0.2)	2 (0.4)	0.506
Laboratory variables			
NT-proBNP, pg/mL (IQR)	159.6 (55.1–627.0)	155.1 (44.1–554.2)	0.578
eGFR, min/1.73 m^2^ (IQR)	45.5 (37.8–55.1)	56.1 (41.9–59.0)	0.004
Outcome variables			
Death, *n* (%)	41 (6.6)	43 (7.7)	0.547
Re-PCI, *n* (%)	41 (6.6)	41 (7.3)	0.323

Categorical data are presented as counts and percentages and analyzed using Chi-square-test. Continuous data are presented as median and the respective interquartile range and analyzed using the Mann-Whitney U test. ST-elevation myocardial infarction (STEMI); Chronic kidney disease (CKD); Myocardial infarction (MI); Transient ischemic attack (TIA); Non-vitamin K antagonist oral anticoagulant (NOAC); Vitamin-K antagonist (VKA); N-terminal pro b-type natriuretic peptide (NT-proBNP); Estimated glomerular filtration rate (eGFR); Re- percutaneous coronary intervention (re-PCI).

**Table 4:** Cox proportional hazard model for the influence of P2Y_12_ inhibitor loading on the composite endpoint cardiovascular death/re-PCI.

	HR (95% CI)	*p*-Value
Univariate	1.01 (0.86–1.19)	0.869
Multivariate	1.04 (0.88–1.21)	0.669

Adjusted for: Age, Gender, antiplatelet pre-medication, prior MI and prior stroke.

## Data Availability

All data are available upon reasonable request to the corresponding author.
